# SVNN: an efficient PacBio-specific pipeline for structural variations calling using neural networks

**DOI:** 10.1186/s12859-021-04184-7

**Published:** 2021-06-19

**Authors:** Shaya Akbarinejad, Mostafa Hadadian Nejad Yousefi, Maziar Goudarzi

**Affiliations:** Department of Computer Engineering, Sharif University of Technology, Azadi Ave., Tehran, Iran

**Keywords:** Structural variation calling, PacBio, Long reads, Neural networks

## Abstract

**Background:**

Once aligned, long-reads can be a useful source of information to identify the type and position of structural variations. However, due to the high sequencing error of long reads, long-read structural variation detection methods are far from precise in low-coverage cases. To be accurate, they need to use high-coverage data, which in turn, results in an extremely time-consuming pipeline, especially in the alignment phase. Therefore, it is of utmost importance to have a structural variation calling pipeline which is both fast and precise for low-coverage data.

**Results:**

In this paper, we present SVNN, a fast yet accurate, structural variation calling pipeline for PacBio long-reads that takes raw reads as the input and detects structural variants of size larger than 50 bp. Our pipeline utilizes state-of-the-art long-read aligners, namely NGMLR and Minimap2, and structural variation callers, videlicet Sniffle and SVIM. We found that by using a neural network, we can extract features from Minimap2 output to detect a subset of reads that provide useful information for structural variation detection. By only mapping this subset with NGMLR, which is far slower than Minimap2 but better serves downstream structural variation detection, we can increase the sensitivity in an efficient way. As a result of using multiple tools intelligently, SVNN achieves up to 20 percentage points of sensitivity improvement in comparison with state-of-the-art methods and is three times faster than a naive combination of state-of-the-art tools to achieve almost the same accuracy.

**Conclusion:**

Since prohibitive costs of using high-coverage data have impeded long-read applications, with SVNN, we provide the users with a much faster structural variation detection platform for PacBio reads with high precision and sensitivity in low-coverage scenarios.

## Background

The complete catalog of genetic variants can be categorized into single-nucleotide polymorphism (SNP), small insertions, and deletions (indels), and structural variations (SV). Structural variations like insertion, deletion, translocations, inversion, and duplications of size larger than 50bp have been associated with a plethora of human diseases and phenotypic traits [1]. In particular, SVs are shown to contribute to different human diseases, including autism, Down syndrome, Alzheimer’s, and also play a pivotal role in the development of aggressive cancers [2]. Despite their importance, characterization of this type of genetic variation lagged behind other forms of variation, e.g. single-nucleotide polymorphism [3], thus developing new methods is needed.

Currently, SV calling tools mostly use short paired-end reads [4]. The variations can be found by the abnormality in the distance between two reads in a pair, alteration in the coverage of a specific region, or split-mapped reads [5]. However, the most salient disadvantage of short-read methods is their limited ability to span and find large SVs. The situation is exacerbated in repetitive regions of the genome and results in the lack of sensitivity, down to 10% [6], and high false discovery rate, up to 89% [7], in short-read tools. On the other hand, long reads are more confidently mapped to repetitive regions, where structural variations abound and are more likely to span breakpoints [8].

The downside of long-read platforms is high sequencing error, e.g. up to 15% for Pacific Biosciences (PacBio) sequencers [9]. As a result, to identify SV breakpoint coordinates, high-coverage reads are required, which in turn, make the alignment phase extremely time-consuming. Among the most notable long-read aligners such as NGMLR [10], Minimap2 [11], IMOS [12], BWA-MEM [13], and BLASR [14], we found Minimap2 to be the fastest and NGMLR to be the most accurate for SV calling—particularly in finding translocations—though significantly slower than Minimap2. Based on our experiments, other aligners (BWA-MEM, and BLASR) were both slower and less accurate than NGMLR for the purpose of SV calling. After the alignment phase, a specialized long-read SV caller is needed to interpret SV’s footprints in alignment file. At this time, four of the most important tools for SV calling are SVIM [15], Sniffles [10], PBHoney [16] and SMRT-SV [17]. Among these SV calling tools, we found SVIM, and Sniffles to be the best in terms of their accuracy and the variety of types of SVs they can detect.

There are a number of softwares available that integrate both alignment and structural variation detection parts. Nanopype [18] is one of them, which utilizes Minimap2, NGMLR, and GraphMap as the aligner and Sniffles as the SV detection tool. However, these pipelines can only use one of the mentioned tools at a time. We hypothesized that if we intelligently combine tools that are either fast or accurate, we can devise a new pipeline which is both fast and accurate. To achieve this feat, we gave all input reads to Minimap2, which is fast; and then we designed and used a neural network to identify only a subset of reads that are useful for SV detection from numerous features of the output of Minimap2. In the next step, only this subset is given to NGMLR, hence minimizing the hefty computational cost of NGMLR while taking advantage of the information it provides for SV detection tools. The logic behind our hypothesis was first, we investigated that only a small fraction of all reads (less than 1%) are used for SV detection, and second, these reads are usually mapped harder to the reference compared to normal reads and therefore might share some common characteristics which can be leveraged in a learning model. Furthermore, we figured out that if we use SVIM alongside Sniffles as SV caller, the sensitivity will be considerably higher in low-coverage situations without aggravating the false discovery rate.

In this paper, we present SVNN: a pipeline for SV detection that intelligently combines Minimap2 and NGMLR for the mapping phase, and SVIM and Sniffles for the SV calling phase. Consequently, the recall rate of our pipeline is considerably higher in low-coverage situations. Concretely, SVNN is up to 3 times faster than the case when we use NGMLR and Sniffles and shows a maximum of 15% recall improvement compared to the case when we utilize Minimap2 and Sniffles.

The heart of our method is a learning model which detects useful reads for structural variation detection and maps this subset of reads. The concept is not restricted to the tools. We chose these tools as they are today’s state of the arts. However, if more advance tools come for alignment or SV calling in the future, we can incorporate them into our platform by retraining the model.

## Implementation

SVNN implements an efficient and accurate pipeline for the detection of structural variations, including indels, inversions, translocations, and duplications. It takes raw long-reads as input and detects SVs in Variant Call Format (VCF). By mapping all reads with Minimap2, and only a useful fragment of all reads with NGMLR, and then calling SVs with SVIM and Sniffles, we ensured SVNN to be precise and efficient at the same time.

We noted that only 0.7% of reads are split by NGMLR and these are the useful reads for SV detection. In other words, the aligning time of only this 0.7% of reads is necessary for SV detection, and the aligning time of the rest of 99.3% of the reads can be avoided for SV calling, as they do not provide useful information for this purpose. Therefore, we can utilize Minimap2 which is up to 10 times faster than NGMLR to map all reads, and then use a classifier to find a superset of reads that are not split by Minimap2 but would have been split by NGMLR (which we call them “informative reads”), and only align this superset of informative reads by NGMLR. We then feed the output of both NGMLR and Minimap2 to Sniffles and SVIM.

**Fig. 1:**
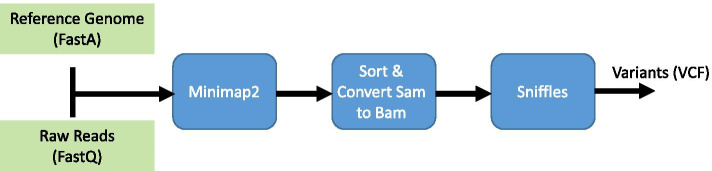
Baseline pipeline

**Fig. 2:**
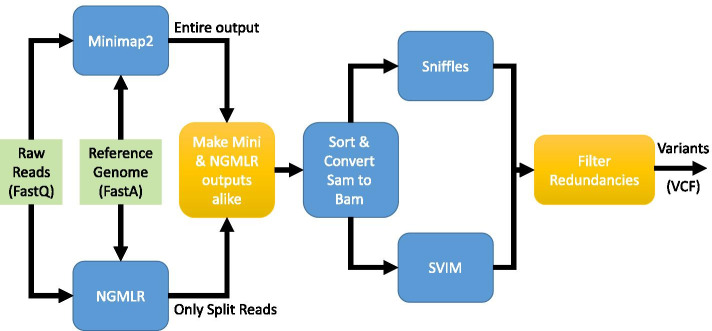
The most sensitive pipeline

**Fig. 3:**
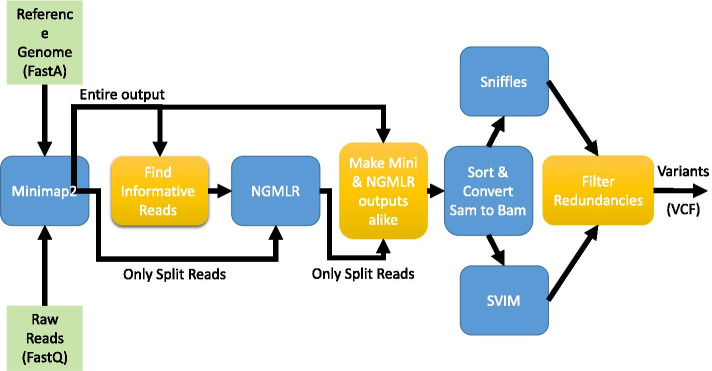
SVNN pipeline

Figure 1 shows the baseline pipeline, which consists of Minimap2 and Sniffles. Figure 2 shows the pipeline of the most sensitive case, which maps all reads by Minimap2 and NGMLR, and then feeds their output to Sniffles and SVIM. In Figs. 2 and 3, golden-colored boxes represent stages that we devised to make the pipeline work. Finally, Fig. 3 shows our pipeline (SVNN), which benefits of every tool while minimizing the overheads. In this pipeline, we first align reads by Minimap2, and then by using a neural network, we try to find informative reads and map only this small subset using NGMLR. Moreover, the sensitivity of combining NGMLR and Minimap2 is higher than utilizing each one separately; as a result, SVNN is more accurate than NGMLR, yet remarkably faster. We further observed by incorporating SVIM alongside Sniffles, the final results will be even better; therefore, we adopted both tools to improve the accuracy of SVNN. In the following, the 6 stages of our pipeline are thoroughly explained.

### Stages of the pipeline

Here we describe the different stages of our pipeline, which is presented in Fig. 3.

#### Map with Minimap2

In this stage, raw reads are mapped to the reference genome by Minimap2. Both reads and reference are defined by the user.

#### Find informative reads

In this stage, some features are extracted from the output of the previous step. Subsequently, these features are fed to a pre-trained neural network to detect informative reads. Here, only a small subset of reads is selected to be given to NGMLR.

By exhaustive search through different hyperparameters of the neural network, we chose a neural network with five fully-connected hidden layers with 18, 30, 18, 11, and 5 neurons in each layer, and tanh as the activation function.

To select which features to use for the detection of informative reads, we first took advantage of SAM file tags, specified by Minimap2. These features include:

- s1: Chaining score
- s2: Chaining score of the best secondary chain
- cm: Number of minimizers on the chain
- NM: Total number of mismatches and gaps in the alignment
- AS: Alignment score

However, we saw that these features alone are not suitable indicators for informative read classification. Therefore, we extracted more features ourselves. Since informative reads are the reads that should have been split, we assumed that the alignment of these reads would be noisier compared to other reads. Therefore, we extracted these features:

- Total number of insertions.
- Total number of deletions.
- Total number of mismatches.
- Longest number of deletions, insertions, and mismatches in a row for a read.
- Longest number of deletions, insertions, and mismatches in a row with one match in between.
- Length of soft-clipped at two ends of the read.
- All aligned reads are divided into 100 bp bins. First, second, third, and fourth bins with the most number of insertions, deletions, and mismatches are selected, and their values are returned.
- Length of the read.

It is important to note that these features are location-independent, which means none of these features rely on the location of the reads. Therefore, if our training datasets are prone to have SVs in a specific location in the reference, it will not overfit to that location.

To train our network, we label informative reads 1 and label other reads 0. The goal of the neural network is to classify these reads.

#### Map with NGMLR

In this stage, selected informative reads from the previous step and split reads of Minimap2 are given to NGMLR. Since Minimap2 split some reads wrongly, especially reads that span translocation, Minimap2 split reads are given to NGMLR as well.

#### Unify Minimap2 and NGMLR outputs

As NGMLR and Minimap2 use different tags in the SAM file, we cannot simply combine their outputs; otherwise, samtools raise an error for converting SAM to BAM. As a result, these tags must become the same before combining them. After combining, the SAM file is converted to the BAM file with samtools.

This stage creates redundancy. In particular, a read might be split by both NGMLR and Minimap2. In this case, the SV detection tool would count this read as two separate reads that support an SV.

#### Detect SVs by SVIM and Sniffles

BAM file generated from the last step is given separately to SVIM and Sniffles. This step, too, creates redundancy, as one SV can be detected by both Sniffles and SVIM.

#### Filter redundant SVs

As we saw, steps 4 and 5 create redundancy. To be detected, each SV needs to be supported by a minimum number of supporting reads. When we combine Minimap2 and NGMLR outputs, some SVs might be supported 2 times by a single read. To resolve this, we count the number of unique supporting reads, and if this number is less than the minimum required supporting reads, we remove that SV.

Also, in step 5, an SV might be detected by both Sniffles and SVIM. To resolve this redundancy, those SVs that are of the same type and their difference in breakpoint location is less than 50 bp, are unified, and the SV with more number of supporting reads would be selected.

## Experiments and results

### Preparing training and test data

For training of our neural network, we injected SVs to chromosomes 21 and 22 of hg 19 by SURVIVOR [19]. We generated more than 16 references with SURVIVOR out of the original reference. Each of our references includes either indel, duplation, translocation, or inversion of length 80 to 1kbp, 1k to 10 kbp, 10k to 30kbp, and 30 to 50kb. Then, by PBSIM [20], we generated PacBio-like reads with 20×, 30×, and 50× coverages. In total, we created 30 fastQ files that include PacBio-like reads from 16 references with SVs. Afterward, we mapped these 30 fastQ files with Minimap2 and NGMLR. We labeled reads that were not split by Minimap2 but were split by NGMLR by a ’1’ tag, and others by a ’0’ tag. Reads that were split by Minimap2 were removed because these reads are given to NGMLR anyway, and there is no need for the neural network to learn to classify these reads. We then extracted our desired features from each read and saved them with their corresponding label to a CSV file.

To test the performance of SVNN, we carried out two types of experiments. In the first type, we used the simulated reads mapped to the unchanged reference. We injected more than 800 SVs to chromosomes 21 and 22, 214 deletions, 186 insertions, 100 duplications, 100 inversions, and 200 translocations. Their length varies from 80 to 50kbp. Like before, we created PacBio-like reads with PBSIM, in 8×, 10×, and 12× coverages. To show our method also generalizes to chromosomes other than chromosomes 21 and 22, which the neural network was trained on, we injected 600 SVs, including 198 deletions, 202 insertions, 100 inversions, and 100 duplications to chromosome 1 and generated PacBio-like reads with 12× coverage. We mapped these reads with Minimap2 and extracted features to our trained neural network to classify reads. To evaluate the performance of the neural network on test sets, we mapped these reads with NGMLR as well.

Since simulation cannot precisely imitate real biological data, in the second part of our experiments we utilized the real PacBio reads of the NA12878 dataset. We selected those reads that were mapped to chromosome 21 and 22 with NGMLR, and among them, we sampled reads to produce a 12× coverage subset of the dataset. We injected 10 insertions and 10 deletions to the reference genome and mapped real reads to this altered genome. The task of the structural variation caller in this scenario is to find the inverse variation that was injected.

### Accuracy results

We compared SVNN with three other pipelines: the baseline, which is Minimap2-Sniffles combination, NGMLR-Sniffles combination, and the most sensitive method, which uses both NGMLR and Minimap2 as the aligner, and Sniffles and SVIM as SV caller. We measured two parameters for each pipeline: Sensitivity and False Discovery Rate (FDR). Sensitivity is the ratio of correctly detected SVs to all SVs, and FDR is falsely detected SVs to all detected SVs. An SV is detected correctly if its type is correct, and its breakpoint distance to the original breakpoint is less than 10bp. Multiple reads must confirm the location and type of a structural variation to make sure the SV is called correctly and is not a false positive. The minimum number of those reads, which is identified by the user, is called supporting reads. We considered the minimum supporting reads for Sniffles to be half of the coverage; for example, the minimum supporting reads for 10× coverage is 5. The reason for this choice is that we need at least half of the average depth support that SV.

#### Accuracy of simulated reads

Tables 1, 2 and 3 show the accuracy of different methods with SVs in chromosomes 21 and 22 for 10, 8, and 12× coverage respectively. In 10× coverage, SVNN’s overall sensitivity, compared to baseline, has improved 14.2 percentage points, but FDR increased 1.8 percentage points, which is negligible in comparison to the increase in sensitivity. Moreover, compared to the most sensitive method, SVNN’s sensitivity is only 0.7 percentage points far from that of the most sensitive case, with 1.8 percentage points improved FDR. The most noticeable improvement, compared to the baseline, is seen in deletion with 22 percentage points. Then duplication with 21 percentage points and translocation with 18 percentage points improvement.

**Table 1:** Accuracy of different methods for simulated reads with 10× coverage on chromosomes 21 and 22

Method	DEL recall (%)	INS recall (%)	INV recall (%)	DUP recall (%)	TRA recall (%)	Overall recall (%)	FDR (%)
Baseline method	58.8	41	92	57	57	58	3.2
NGMLR-Sniffles	61.6	14.5	92	60	66.5	55.5	2.5
Most sensitive method	81.3	46.2	95	78	76	73	6.8
SVNN	80.8	46.2	95	78	75	72.2	5

**Table 2:** Accuracy of different methods for simulated reads with 8× coverage on chromosomes 21 and 22

Method	DEL recall (%)	INS recall (%)	INV recall (%)	DUP recall (%)	TRA recall (%)	Overall recall (%)	FDR (%)
Baseline method	62.6	42.9	91	65	54.5	58.25	3.8
NGMLR-Sniffles	58.4	13.9	87	63	62	53.1	3.5
Most sensitive method	84.5	49.4	94	80	78	75.5	9
SVNN	79.4	47.3	94	74	78	72.7	6.1

**Table 3:** Accuracy of different methods for simulated reads with 12× coverage on chromosomes 21 and 22

Method	DEL recall (%)	INS recall (%)	INV recall (%)	DUP recall (%)	TRA recall (%)	Overall recall (%)	FDR (%)
Baseline method	63.3	37.1	92	64	50	57.5	3
NGMLR-Sniffles	62.6	10.2	88	67	63	54.25	3.4
Most sensitive method	85.9	47.8	95	80	76.5	75.1	6.4
SVNN	84.1	46.2	95	77	76.5	73.8	4.6

In 8× coverage (Table 2), SVNN is 14 percentage points more sensitive than the baseline pipeline. Like before, the most impressive progress is seen in deletion and translocation detection. Furthermore, due to the fewer number of supporting reads, the sensitivity of SVNN did not change very much compared to the most sensitive method, but FDR reduced to 6.1%.

In 12× coverage, most improvement is seen in translocation with a 26% difference. Also, compared to the baseline, we had 16.3 percentage points improvement in sensitivity and a 1.6 percentage point increase in FDR. Compared to the most sensitive method, we experienced only a 1.3 percentage point reduction in sensitivity but a 1.8 percentage point improvement in FDR.

We can observe from these tables that for calling some SVs, like translocations, NGMLR works best (for alignment phase) and, for calling some others, like insertions, Minimap2 works best.

**Table 4:** Accuracy of different methods for simulated reads with 12× coverage on chromosome 1

Method	DEL recall (%)	INS recall (%)	INV recall (%)	DUP recall (%)	Overall recall (%)	FDR (%)
Baseline method	71.2	41.1	92	68	64	3.4
NGMLR-Sniffles	67.7	13.8	84	70	52.6	2.8
Most sensitive method	85.8	49.5	95	90	75.5	4.6
SVNN	83.3	45.5	95	88	73.3	4

Table 4 shows the result of different methods on chromosome 1. Here, SVNN’s sensitivity is 9.3 percentage points better compared to the baseline method. The gain in recall rate is especially seen in the detection of deletions and duplications with 12 and 10 percentage points respectively.

The results on chromosome 1 show that the model is not overfitted to the location of reads, or the chromosome they are generated from. Our model was trained based on reads from chromosomes 21 and 22, however, it worked properly to classify informative reads on chromosome 1 too.

It is evident from Tables 1, 2, 3 and 4 that all these methods are least sensitive in detecting insertions.

#### Accuracy of real reads

Table 5 depicts the accuracy of four different pipelines when the reference is altered and reads are real with 12× coverage. 10 insertions were simulated by deleting 10 regions of chromosomes 21 and 22, and 10 deletions were simulated by inserting a random sequence to different locations of the reference. Since in this case, duplications are treated like deletions, they cannot be simulated. Unfortunately, regarding these PacBio reads, neither Sniffles nor SVIM worked accurately for detecting inversions and translocations. Therefore, we avoided adding them to the reference. Among the simulated SVs, SVNN worked 10 percentage points better than Minimap2-Sniffles, 20 percentage points better than NGMLR-Sniffles, and was on par with the most sensitive method.

**Table 5:** Accuracy of different methods for real reads with 12× coverage on chromosomes 21 and 22

Method	DEL recall (%)	INS recall (%)	Overall recall (%)
Baseline method	70	60	65
NGMLR-Sniffles	70	40	55
Most sensitive method	80	70	75
SVNN	80	70	75

### Speed results

In Tables 6, 7, 8, 9 and 10, the running time for each phase is described in each column. All times are in seconds. These times are derived from running each task on a computer with an Intel Core i7 quad-core CPU and 16 GigaBytes of RAM. The last column of these tables shows the speed-up of SVNN compared to the method in that row. For example, when 1.98 is written in the speed-up column, it means the ratio of running time of this method to SVNN is 1.98, or in other words, SVNN is 1.98 times faster than this method.

#### Speed analysis of simulated reads

**Table 6:** Running time of different methods for simulated reads with 10× coverage on chromosomes 21 and 22

Method	Time (s)									
	Minimap2	NGMLR	Find reads in fastQ	Unify outputs	SAM to BAM	Sniffles	SVIM	Remove redundancy	Total	Speed-up
Baseline method	204	–	–	–	61	37	–	–	302	0.25
NGMLR-Sniffles	–	1961	–	–	50	34	–	–	2045	1.74
Most sensitive method	204	1961	–	28	65	37	43	2	2340	1.98
SVNN	204	665	146	21	64	37	40	2	1179	1

**Table 7:** Running time of different methods for simulated reads with 8× coverage on chromosomes 21 and 22

Method	Time (s)									
	Minimap2	NGMLR	Find reads in fastQ	Unify outputs	SAM to BAM	Sniffles	SVIM	Remove redundancy	Total	Speed-up
Baseline method	163	–	–	–	47	27	–	–	233	0.24
NGMLR-Sniffles	–	1586	–	–	42	25	–	–	1653	1.68
Most sensitive method	163	1586	–	16	50	29	35	2	1881	1.91
SVNN	163	590	104	13	50	27	32	2	981	1

**Table 8:** Running time of different methods for simulated reads with 12× coverage on chromosomes 21 and 22

	Time (s)									
Method	Minimap2	NGMLR	Find reads in fastQ	Unify outputs	SAM to BAM	Sniffles	SVIM	Remove redundancy	Total	Speed-up
Baseline method	281	–	–	–	78	72	–	–	431	0.28
NGMLR-Sniffles	–	2687	–	–	66	67	–	–	2820	1.84
Most sensitive method	281	2687	–	51	119	77	51	2	3266	2.14
SVNN	281	766	196	43	117	74	50	2	1529	1

Tables 6, 7 and 8 show the running time of all methods in 10, 8 and, 12× coverage, with SVs located in chromosomes 21 and 22. Table 9 shows the running time of all methods in 12× coverage executed on chromosome 1. By observing Tables 1 and 6, we see that in 10× coverage, SVNN only lacks 0.8 percentage points in sensitivity than the most sensitive method, but it is 2 times faster. In this coverage, SVNN’s sensitivity is 16.7 percentage points more than NGMLR-Sniffles, and its running time is 1.74 times less.

In Table 8, in 12× coverage, SVNN is 1.84 times faster than NGMLR-Sniffles and sensitivity improved 19.55 percentage points. In this coverage, the speed compared to the most sensitive method amazingly got 2.1 times faster.

Nowhere in our experiments, was the speed-up as high as in Table 9. The speed-up compared to the most sensitive method and NGMLR-Sniffles is 3.6 and 3.1, respectively. The reason for this higher speed-up is that the SVs were injected in a single chromosome, and therefore, translocations could not be simulated. Not having translocations made the learning model much more accurate, and a smaller subset of reads was selected to be mapped by NGMLR.

**Table 9:** Running time of different methods for simulated reads with 12× coverage on chromosome 1

	Time (s)									
Method	Minimap2	NGMLR	Find reads in fastQ	Unify outputs	SAM to BAM	Sniffles	SVIM	Remove redundancy	Total	Speed-up
Baseline method	556	–	–	–	203	130	–	–	889	0.48
NGMLR-Sniffles	–	5446	–	–	202	123	–	–	5771	3.14
Most sensitive method	556	5446	–	105	346	142	147	1	6743	3.66
SVNN	556	278	439	97	205	128	134	1	1838	1

**Table 10:** Running time of different methods for real reads with 12× coverage on chromosomes 21 and 22

	Time (s)									
Method	Minimap2	NGMLR	Find reads in fastQ	Unify outputs	SAM to BAM	Sniffles	SVIM	Remove redundancy	Total	Speed-up
Baseline method	838	–	–	–	82	41	–	–	961	0.27
NGMLR-Sniffles	–	3577	–	–	86	60	–	–	3723	1.04
Most sensitive method	838	3577	–	91	142	98	85	10	4841	1.35
SVNN	838	1780	561	87	132	96	81	9	3584	1

#### Speed analysis of real reads

Our learning model was trained on simulated reads by PBSIM, it had never seen examples of real reads. Therefore, its specificity dropped significantly for real reads. About 50% of reads were identified as informative reads. As a result, more reads were given to NGMLR, and the program took much longer to run. Despite the difference between the training and test sets, SVNN is 1.35 times faster than the most sensitive method and 1.04 times faster than NGMLR-Sniffles. This means on real data, which our model has not seen, SVNN achieves 20 percentage points improvement in accuracy compared to NGMLR-Sniffles and still finishes its task faster.

Another point we see in real data is that almost all steps took longer to run compared to 12x synthetic reads in Table 8. The main reason for this observation is that real reads were on average longer than synthetic reads. Average length of real reads was 4500 bps, while average synthetic read length was 2800 bps.

## Conclusions

In this paper, we have presented SVNN: an SV detection pipeline that leverages both the speed of Minimap2 and the accuracy of NGMLR for SV calling. Our main aim in this work had been devising a tool for SV detection which can be fast while maintaining high sensitivity. Not only did we achieve this feat, but we also were able to increase the sensitivity up to 20 percentage points. SVNN’s high recall rate and low false discovery, make it suitable for low-coverage situations, thus even further minimizing the cost of long-read applications. SVNN is available for Linux and has a user-friendly interface. The user only needs to specify raw reads, the reference, and the number of supporting reads; SVNN takes care of the rest. SVNN can also be extended to include other tools. By retraining another network, new tools can be integrated together. This paper is written as a proof-of-concept to show that for an end-to-end bioinformatics application like SV calling, we can use a learning model to combine the plus sides of existing tools, and considerably improve their performance.

In the future, we can improve the performance of SVNN in two directions: speed-wise, and FDR-wise. Since NGMLR is still the computational bottleneck of SVNN, we can speed up the whole process by multi-node implementation or parallel implementation of NGMLR on GPU. On the other hand, as we saw, FDR increases when we use Minimap2 for alignment. The reason is that some reads are wrongly split by Minimap2. Hence, another neural network can be trained to classify the wrongly split reads and reduce the FDR.

Furthermore, since SVNN was trained on PacBio-like reads, it currently is not suitable for Oxford Nanopore reads. We plan to train another model for the Nanopore reads, and provide users with two separate models which they can choose from based on what type of reads they have: PacBio or Nanopore.

## Availability and requirements

*Project name* SVNN

*Project home page* http://easy.ce.sharif.edu/svnn/

*Operating system(s)* Ubuntu

*Programming language* Python and C++

*Other requirements* Python 3.5 or higher, G++ 11 or higher

*License* CC BY 4.0

*Any restrictions to use by non-academics* None.

## Data Availability

Source codes and a detailed manual are freely available at http://easy.ce.sharif.edu/svnn/.

## Abbreviations

SNP: Single-nucleotide polymorphism
SV: Structural variation
PacBio: Pacific Biosciences
DEL: Deletion
INS: Insertion
DUP: Duplication
INV: Inversion
INDEL: Insertion and Deletion
INVDEL: Inversion flanked with deletion
INVDUP: Inverted duplications
VCF: Variant Call Format
